# Development of a rapid on-site detection method for largemouth bass virus based on RPA-CRISPR/Cas12a system

**DOI:** 10.3389/fmicb.2025.1599006

**Published:** 2025-05-21

**Authors:** Na Li, Chunmei Dai, Yuelin Mao, Yimeng Zhang, Huiyuan Yan, Huijiao Wang, Yinghui Qin, Baicheng Huang, Xinjie Wang, Lunguang Yao

**Affiliations:** ^1^Henan Provincial Engineering and Technology Center of Health Products for Livestock and Poultry, Henan Field Observation and Research Station of Headwork Wetland Ecosystem of The Central Route of South-to-North Water Diversion Project, School of Life Science, Nanyang Normal University, Nanyang, China; ^2^Zhejiang Lab, Hangzhou, China; ^3^Genome Analysis Laboratory of the Ministry of Agriculture and Rural Affairs, Agricultural Genomics Institute at Shenzhen, Chinese Academy of Agricultural Sciences, Shenzhen, China

**Keywords:** largemouth bass virus, *MCP* gene, recombinase polymerase amplification, CRISPR/Cas12a, detection

## Abstract

**Introduction:**

Largemouth bass virus (LMBV), the causative agent of largemouth bass ulcerative syndrome, poses a significant economic threat to the aquaculture industry. Rapid, simple, and reliable detection methods are essential for the timely identification of LMBV infections, enabling effective prevention and control measures.

**Methods:**

In this study, a detection platform utilizing the clustered regularly interspaced short palindromic repeats (CRISPR)/CRISPR-associated protein 12a (Cas12a) system was developed for LMBV. CRISPR RNA (crRNA) and recombinase polymerase amplification (RPA) primers were designed to target the highly conserved region of the major capsid protein (*MCP*) gene. Additionally, a one-pot method and lyophilization strategy were optimized for field applications.

**Results:**

The RPA-CRISPR/Cas12a system achieved a sensitivity of 50 copies/reaction within 40 minutes, without requiring specialized equipment, and exhibits high specificity for LMBV. Validation with 42 clinical samples of suspected LMBV infection demonstrated 100% concordance among the RPA-CRISPR/Cas12a method, quantitative PCR and lateral flow strip assay. The one-pot method and lyophilization strategy demonstrated consistent detection results with two-step RPA-CRISPR methods in clinical sample testing, offering more convenient and stable application characteristics for on-site detection.

**Discussion:**

This study establishes an efficient process for detecting LMBV nucleic acids in fish clinical samples, culminating in a CRISPR-based fluorescent readout, offering significant advantages for viral diagnosis and monitoring.

## 1 Introduction

Largemouth bass (*Micropterus salmoides*) is a globally significant and economically important freshwater fish (Wang et al., 2019). In recent years, it has become a dominant species in aquaculture in China (Harimana et al., 2019). However, the expansion of aquaculture and the associated high stocking densities have exacerbated disease challenges, particularly from viral pathogens (Deng et al., 2010; Ma et al., 2013; Chen et al., 2015; Sibley et al., 2016). Among these, largemouth bass virus (LMBV), a nucleocytoplasmic large DNA virus of the genus *Ranavirus* (family *Iridoviridae*), poses one of the most serious threats to the global largemouth bass aquaculture industry (Huang et al., 2014; Jia et al., 2020). LMBV infections can result in clinical signs such as splenomegaly, renomegaly, severe skin ulcerations, and necrosis of muscle tissue, often culminating in high mortality rates and substantial economic losses (Zilberg et al., 2000; Blazer et al., 2010).

Early and rapid diagnosis of LMBV is critical for effective disease control. Conventional polymerase chain reaction (PCR) (Grizzle et al., 2003; Jeong et al., 2006; Iwanowicz et al., 2013) and quantitative PCR (qPCR) (Getchell et al., 2007; Groocock et al., 2008) are widely used diagnostic methods but are limited by complex protocols and the need for expensive equipment. As a result, there is an urgent need for faster, more affordable, and field-adaptable diagnostic methods for LMBV in fish farms.

CRISPR-based detection systems have recently gained attention for their exceptional specificity, sensitivity, and programmability, making them well-suited for nucleic acid detection (Chen et al., 2018; Broughton et al., 2020). Within the CRISPR system, Cas12a has emerged as a reliable tool for DNA detection due to its *cis-* and *trans-*cleavage activities (Li et al., 2018). The CRISPR/Cas12 system offers high specificity and rapid reaction times, making it ideal for precise pathogen identification (Zhao et al., 2023). The most commonly used Cas12a enzymes, *Acidaminococcus* sp. Cas12a (AsCas12a) and *Lachnospiraceae bacterium* Cas12a (LbCas12a), have been applied in the diagnosis of many infectious diseases (Li et al., 2022a; Sui et al., 2022). Extensive studies have demonstrated that AsCas12a exhibits superior performance over LbCas12a in detection applications, particularly in terms of specificity, temperature stability, and detection efficiency (Kim et al., 2016; Chen et al., 2018; Broughton et al., 2020). Based on these advantages, AsCas12a was selected for this study.

RPA is a novel isothermal amplification technique characterized by rapid reaction times (at 37–42°C) and minimal equipment requirements (Dai et al., 2019). Combining CRISPR with isothermal amplification methods has led to the development of innovative nucleic acid detection techniques (Gootenberg et al., 2017). These approaches leverage the precision of CRISPR systems to deliver rapid, highly sensitive, specific, and cost-effective diagnostic solutions (Wang et al., 2016). RPA combined with Cas12a has been successfully applied to detect various pathogens, including human immunodeficiency virus type 1 (HIV-1) (Zhao et al., 2021), goose astrovirus (GAstV) (Yang K. et al., 2022), Noroviruses (NoVs) (Fang et al., 2023), porcine circovirus type 3 (PCV3) (Jiang et al., 2024), *Vibrio vulnificus* (Zhang et al., 2023), *Klebsiella pneumoniae* (Tan et al., 2024), *Candida albicans* (Liu R. et al., 2024), and *Fasciola hepatica* (Yang et al., 2024).

In this study, we aimed to establish a feasible, specific, sensitive, and reliable RPA-CRISPR/Cas12a-based visual detection method for rapid detection of LMBV. To achieve this, we compared the major capsid protein (*MCP*) gene sequences of LMBV to identify optimal crRNA and RPA primers for CRISPR-based detection. This method provides significant advantages for the clinical diagnosis and monitoring of LMBV.

## 2 Materials and methods

### 2.1 Pathogens and clinical samples

The complete coding sequence of major capsid protein (*MCP*) gene from a reference strain LMBV-FS2021 (GenBank accession: ON418985.1) was selected for bioinformatics analyses. The eight pathogens, including *Aeromonas veronii* (*A. veronii*), *Streptomyces xiamenensis* (*S. xiamenensis*), *Plesiomonas shigelloides* (*P. shigelloides*), *Citrobacter freundii* (*C. freundii*), *Micropterus salmoides* rhabdovirus (MSRV), Red spotted grouper nervous necrosis virus (RGNNV), Snakehead vesiculovirus (SHVV) and Grass Carp Reovirus (GCRV), were used in the specificity assay.

In this study, a total of 42 clinical samples of fish liver (samples #1 to #42) with clinical characteristics were collected at a commercial fish farm in Nanyang, Henan Province, China. The primary clinical symptoms of the diseased fish exhibited ulcer lesions and mild bleeding appeared on the body surface. Among these samples, 19 samples tested positive and 23 samples tested negative for LMBV by qPCR (Novoprotein, China) (Supplementary Table S1).

### 2.2 *MCP* gene and phylogenetic analysis

The plasmid containing the full-length *MCP* gene of LMBV-FS2021 (GenBank accession: ON418985.1) (Supplementary Table S2) was kindly provided by Dr. Qin (Qin et al., 2024). *MCP* gene conservation was assessed across all 19 sequences obtained from GenBank using the query: “((LMBV[Title]) OR Largemouth Bass Virus[Title]) AND COMPLETE.” Phylogenetic analysis was performed using the neighbor-joining algorithm in MEGA X software. Multiple sequence alignment was generated using MUSCLE, and tree robustness was evaluated with 1,000 bootstrap replicates. Viral strains analyzed in this study, including their information and GenBank references are presented in Supplementary Table S3.

### 2.3 RPA reaction

In the RPA procedure, a kit (GenDx Biotech, China) was employed following the report with slight adjustment (Huang et al., 2024). Briefly, the reaction solution was assembled in a final volume of 24 μL, comprising 10 μL of reaction buffer, 5.0 μL of ERA buffer, 1.5 μL of forward primer (10 μM), 1.5 μL of reverse primer (10 μM), 3.0 μL of template DNA (concentration adjusted according to specific applications), and 3.0 μL of ddH_2_O. The mixture was vortexed briefly and centrifuged prior to the addition of 1.0 μL of activator (magnesium acetate, 200 mM). The reaction was then incubated at 39°C for 20 min. The RPA products were subsequently used for downstream applications.

### 2.4 CRISPR/Cas12a reaction system

Within a 20 μL CRISPR assay, the reaction solution of 15 μL (excluding the template) was assembled by combining the components as follows: 8.8 μL ddH_2_O, 2 μL reaction buffer, 0.2 μL RNase inhibitor (Novoprotein, China), 1 μL FAM-ssDNA-BHQ1 reporter (Genewiz, China), 2 μL crRNA (1 μM, synthesized by Genscript, China), 1 μL engineered enAsCas12a-Ultra protein (enAsU-R). Subsequently, 5 μL RPA product was added into the CRISPR reaction tube. The mixture was then subjected to incubation at 37°C for 20 min. Following this, fluorescence signals were captured using an LED Transilluminator (Sangon, China). For accurate quantification of fluorescence, the cleavage results of the CRISPR reaction represented by the fluorescence intensity were obtained using a Varioskan^®^ LUX multimode microplate reader (Thermo Scientific, United States). The excitation wavelength was set to 485 nm, and fluorescence emission was collected at 535 nm (Huang et al., 2023; Huang et al., 2024).

### 2.5 Design of crRNA and RPA primers

The crRNA candidates of LMBV *MCP* gene (Table 1), each 42 nucleotides (nt) in length, were designed following the principles of crRNA design (motif of TTTN) and EasyDesign (Huang et al., 2024). The crRNA screening was performed using the DNA template of the *MCP* gene with a Cas12a-based method over a 60-min fluorescence recording period. The crRNA with the highest reactivity was selected for subsequent RPA primer screening. RPA primer pairs were designed following the guidelines provided by the TwistAmp^™^ Basic Kit (TwistDx, United States) and the EasyDesign online design tool. The main design principles include the following: The GC content of the primers is between 35 and 60%; No more than 4 consecutive base forms of A, T, C, and G within the primer; No G within the 3 nt interval at the 5′ terminal of the primer; The primers contain G or C within the 3 nt interval at the 3′ terminal (Huang et al., 2024). The RPA primer candidates for optimal primer screening are listed in Table 2.

**Table 1:** The sequence of crRNAs of LMBC *MCP* gene.

crRNA	Sequence (5′-3′)
*MCP*-cr1	UAAUUUCUACUAAGUGUAGAUACGAAAUAAGUAGUUGCAUCU
*MCP*-cr2	UAAUUUCUACUAAGUGUAGAUGUCAAAGAGCAUUAUCCCGUG
*MCP*-cr3	UAAUUUCUACUAAGUGUAGAUGUAAACCAACCCACGGGAUAA
*MCP*-cr4	UAAUUUCUACUAAGUGUAGAUGGCAGAGACAGCGGGCUGGCC
*MCP*-cr5	UAAUUUCUACUAAGUGUAGAUUAGCCAGAGUUGUCUGCCCCU

**Table 2:** RPA primer candidates for screening optimal primers for LMBV detection.

Primer	Sequence (5′-3′)
*MCP*-F1	GTTCATTGATCTCGCCACTTATGACAGCCT
*MCP*-R1	TGTGGCAGCCGTAGGCAGTTTGGTAAACCA
*MCP*-F2	GCATCACTAGCGGGTTCATTGATCTCGCCA
*MCP*-R2	GAAGTTTTTGTGGCAGCCGTAGGCAGTTTG
*MCP*-F3	GTTCTGGCATCACTAGCGGGTTCATTGATC
*MCP*-R3	TGCTGCCCGAAAGCAGGCGTACCAGAAGTT

### 2.6 qPCR assay

The LMBV DNA was detected by real-time qPCR assay. DNA was isolated from approximately 30 mg dry homogenized liver tissue. All DNA isolation was done using the RNA/DNA nucleic acid extraction kit (Bioer Technology, China) according to manufacturer’s protocol. The DNA concentration and purity were determined by NanoDrop ONE (Thermo Scientific, United States). Simply, the reaction system (NovoStart^®^ SYBR qPCR SuperMix Plus, Novoprotein, China) contained 2 × qPCR SYBR Green Master Mix (10.0 μL), 10 μM forward primer (0.4 μL), 10 μM reverse primer (0.4 μL), 50 × ROX Reference Dye 1 (0.4 μL), and template cDNA (2.0 μL), and ddH_2_O was added to a total reaction volume of 20 μL. Then qPCR was performed on the CFX96 Duet Real-Time PCR system (Bio-Rad, United States). The following thermocycling conditions were used for qPCR: 3 min pre-incubation at 95°C, followed by 40 cycles of 95°C for 5 s; 60°C for 30 s. The threshold cycle (Ct) value of the samples was quantified by qPCR using a pair of *MCP* gene-specific primers. The target region and primer sequences for qPCR detection of LMBV MCP gene are provided in Supplementary Table S4. The samples were defined as positive if its Ct value was ≤33. Conversely, samples with a Ct value > 33 or undetectable Ct values were defined as negative.

### 2.7 Specificity and sensitivity assay

In the specificity assay, 8 pathogens, including *A. veronii*, *S. xiamenensis*, *P. shigelloides*, *C. freundii*, MSRV, RGNNV, SHVV, and GCRV, were tested using the RPA-CRISPR/Cas12a method, all the DNA templates were normalized to 10^8^ copies per reaction. In the sensitivity assay of LMBV, a gradient dilution of the DNA templates was tested, including 10^8^, 10^7^, 10^6^, 10^5^, 10^4^, 10^3^, 10^2^, 50, 10, and 0 copies per reaction. The limit of detection for LMBV was determined based on the fluorescence signal obtained from the RPA-CRISPR/Cas12a detection.

### 2.8 Lateral flow strip assay

Lateral flow strip assay (LFA) was carried out as previously described (Liu Y. et al., 2024) by using commercially available detection strips (GenDx Biotech, China). The CRISPR/Cas12a reaction system for LFA utilized biotin-ssDNA-FAM in place of FAM-ssDNA-BHQ1, while all other components remained unchanged. The strips were designed to detect amplicons dual-labeled with FAM and biotin, and the results were visually identified following the manufacturer’s instructions. For the assay, 20 μL of the Cas12a reaction mixture was added to a tube containing 80 μL of HybriDetect Assay buffer. The mixture was vortexed and centrifuged, after which a lateral flow dipstick was inserted and incubated at room temperature for 2 min. Following incubation, the dipstick was removed and imaged using a smartphone camera.

### 2.9 Clinical sample testing

**Figure 1:**
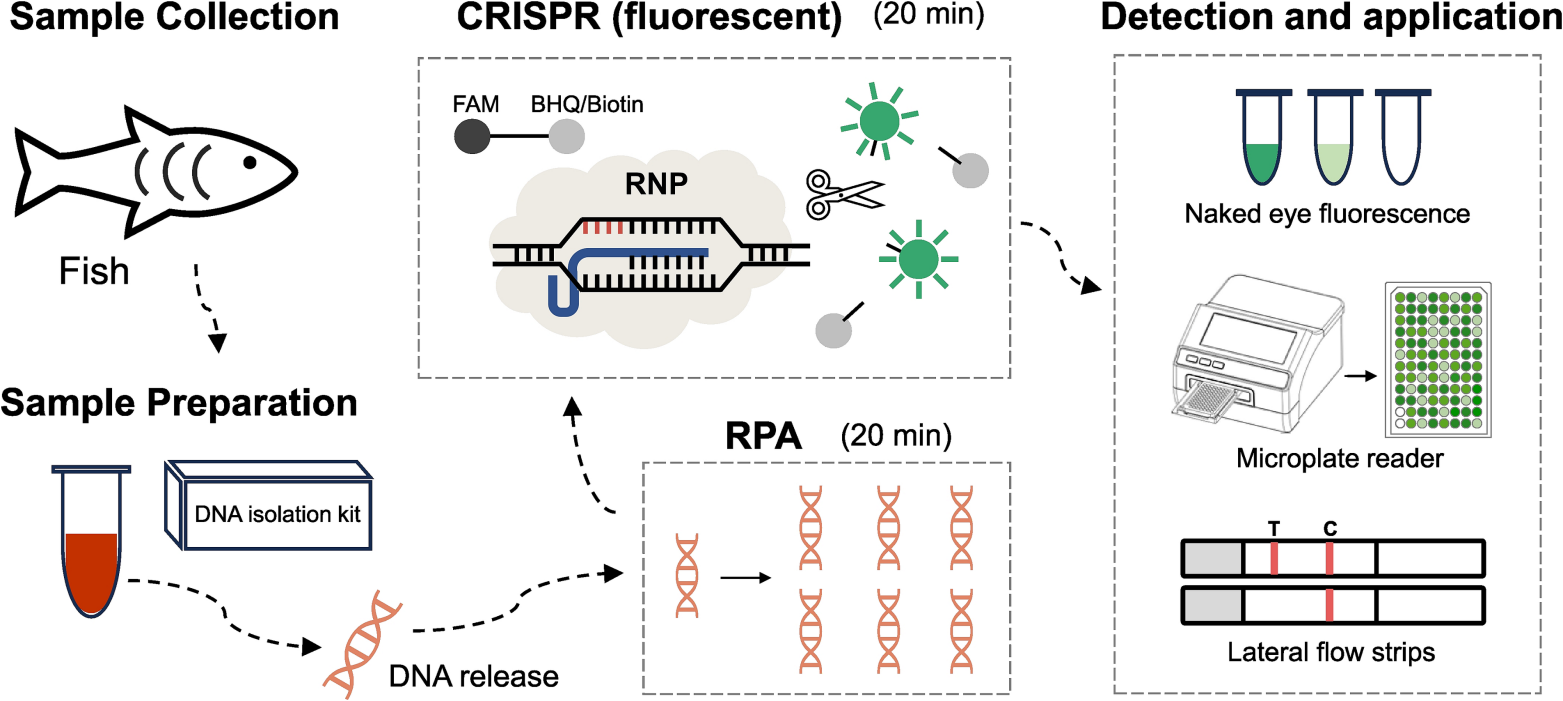
Development of the RPA-CRISPR/Cas12a based method for LMBV detection in fish. Step 1: Collection of samples from fish. Step 2: DNA extraction from fish samples using a DNA isolation kit. Step 3: RPA reaction for target amplification. Step 4: CRISPR reaction for specific detection. Step 5: Fluorescence signal collection using three methods: naked-eye observation, microplate reader detection, and lateral flow strip detection.

A total of 42 clinical samples were analyzed using the established RPA-CRISPR/Cas12a assay. As illustrated in Figure 1, the RPA-CRISPR/Cas12a assay for LMBV detection, involved the following steps: DNA was first extracted from fish-derived samples. The extracted DNA was then subjected to detection of LMBV using the RPA-CRISPR/Cas12a assay described above, employing the optimized crRNA and RPA primers. Results were obtained through three approaches: visual inspection, fluorescence quantification (using a microplate reader), and lateral flow strip analysis. The recombinant pET28-MCP plasmid served as the positive controls (Pos), while ddH_2_O was used as the negative control (Neg). In the absence of the target nucleic acid sequence, only the control line (C) is visible, while the test line (T) remains invisible.

### 2.10 One-pot RPA-CRISPR assay

The one-pot RPA-CRISPR assay combines RPA with CRISPR/Cas12a detection in a single-tube reaction. The RPA reaction was prepared at the bottom of the tube, with the following components: 10 μL of RB reaction buffer, 4 μL ERA buffer, 1.25 μL each of forward and reverse (10 μM), 1 μL of DNA template, 1.0 μL of magnesium acetate (200 mM), and ddH_2_O was added to a total final volume of 20.0 μL. The CRISPR/Cas12a detection, a 5 μL mixture was prepared in tube cap, containing 1 μL of enAsU-R Cas12a protein (200 ng/μL), 1 μL of crRNA (20 μM), 1 μL of FAM-ssDNA-BHQ1 reporter (1 μM), 0.1 μL of RNase inhibitor (40 U/μL), 1.9 μL of ddH_2_O. The reaction was performed at 39°C for 10 min for RPA amplification. Following amplification, the CRISPR/Cas12a reaction system was manually mixed with the RPA amplicons by gentle tube inversion (5–10 times) and incubated at 39°C for 30 min.

### 2.11 Lyophilized reagents for the RPA-CRISPR reaction system

The lyophilized reagents in the reaction tube for the RPA-CRISPR system include all components except the RPA activator (magnesium acetate) and the target DNA sample. Tubes were briefly centrifuged and stored at −80°C until ready for lyophilization overnight (FreeZone^®^ Fully Automatic Top-Loading Capping System, LABCONCO, United States). After lyophilization, the tubes are stored at −20°C or 4°C. For detection, the RPA activator, sample DNA, and ddH_2_O are added directly to the reaction tube containing the lyophilized pellet. The mixture should be thoroughly mixed before incubation at 39°C for 40 min. Fluorescence signals were then captured using an LED Transilluminator (Sangon, China).

### 2.12 Data analyses

To ensure the precision and dependability of the experimental outcomes, each test was conducted in triplicate and subjected to statistical evaluation using GraphPad Prism 8.0. The results are presented as mean ± SEM, and an analysis of ANOVA was performed to assess the differences between experimental group and control group. Statistical significance was defined as a *p* value of <0.05.

## 3 Results

### 3.1 crRNA design and selection based on the LMBV *MCP* gene

**Figure 2:**
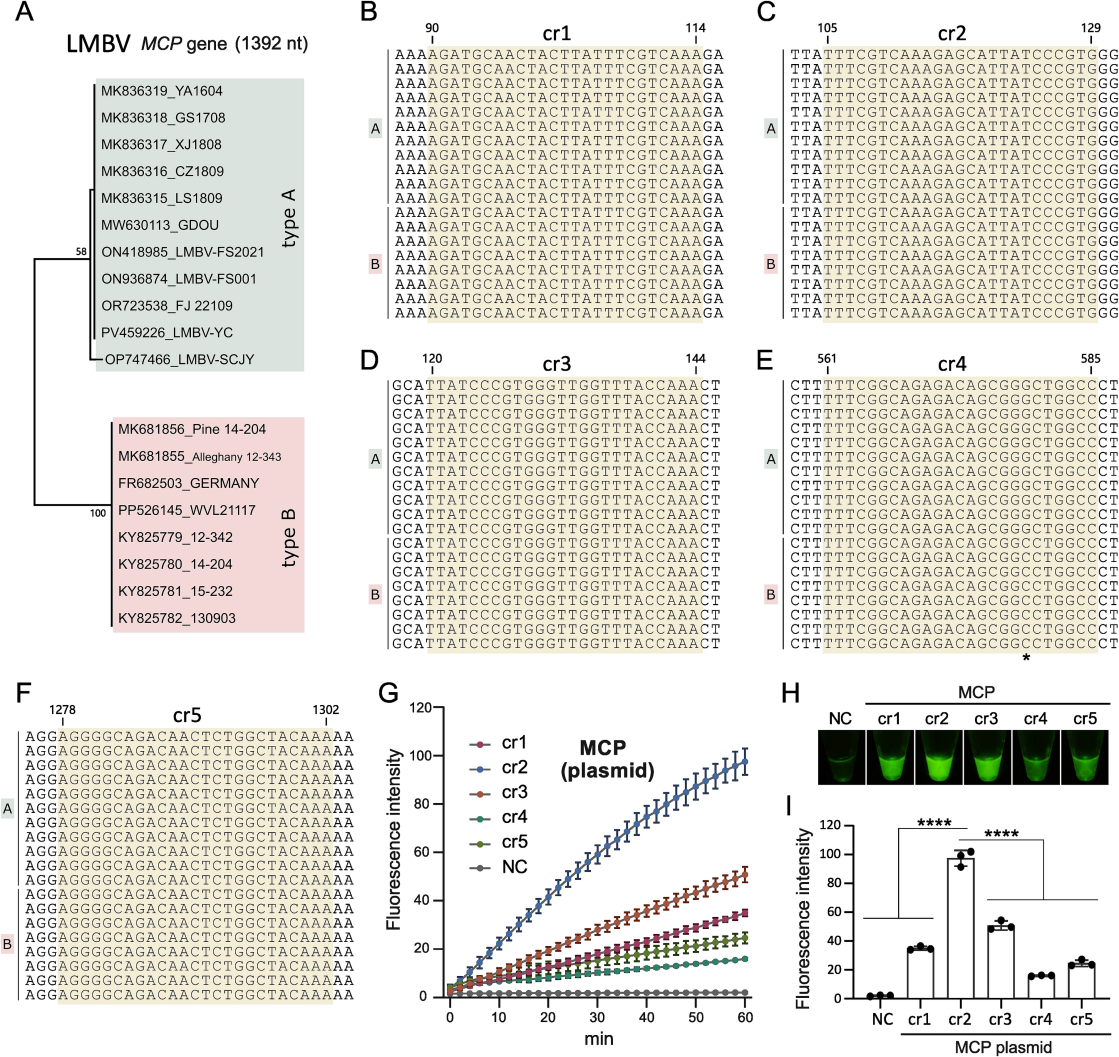
Screening of crRNAs. **(A)** Phylogenetic tree of the LMBV *MCP* gene, constructed using 19 *MCP* gene sequences from GenBank. **(B–F)** Locations of crRNAs of cr1-5 on the target *MCP* gene of LMBV, respectively. The precise location of the crRNAs is indicated, and “*” in **(E)** represents differential site between LMBV type A and type B. (**G**) Efficiency screening of crRNAs over 60 min. **(H)** Fluorescence detection of cr1 to cr5 targeting the *MCP* gene using the CRISPR assay. **(I)** Statistical analysis of cr1 to cr5 performance in the CRISPR assay. NC represents the no-template controls. At least three technical replicates were performed for each biological sample. Error bars represent the standard error of three replicates. ****p < 0.0001.

To improve the detection of clinical LMBV infections, selecting highly active crRNA is essential for the Cas12a-based fluorescence assay. Phylogenetic analysis of available sequences revealed two major types of LMBV *MCP* genes (Figure 2A), type A and type B, differing by 12 nucleotide (nt) out of the 1,392-base segment. Based on these sequences, 5 crRNA candidates (cr1-cr5) targeting the conserved regions of LMBV *MCP* gene were designed (Table 1, Figures 2B–F), with the nt location of 90–114, 105–129, 120–144, 561–585, and 1,278–1,302 in the coding sequence of *MCP* gene, respectively.

During crRNA screening, template DNA representing LMBV *MCP* sequences (Supplementary Table S2) was tested in Cas12a based fluorescence activity assays with the five crRNAs. Among these, *MCP*-cr2 exhibited the highest fluorescence activity in the results of the microplate reader (Figures 2G,I) and the direct observation (Figure 2H), and then was selected as the target crRNA for subsequent experiments.

### 3.2 RPA primer screening based on the selected crRNAs

**Figure 3:**
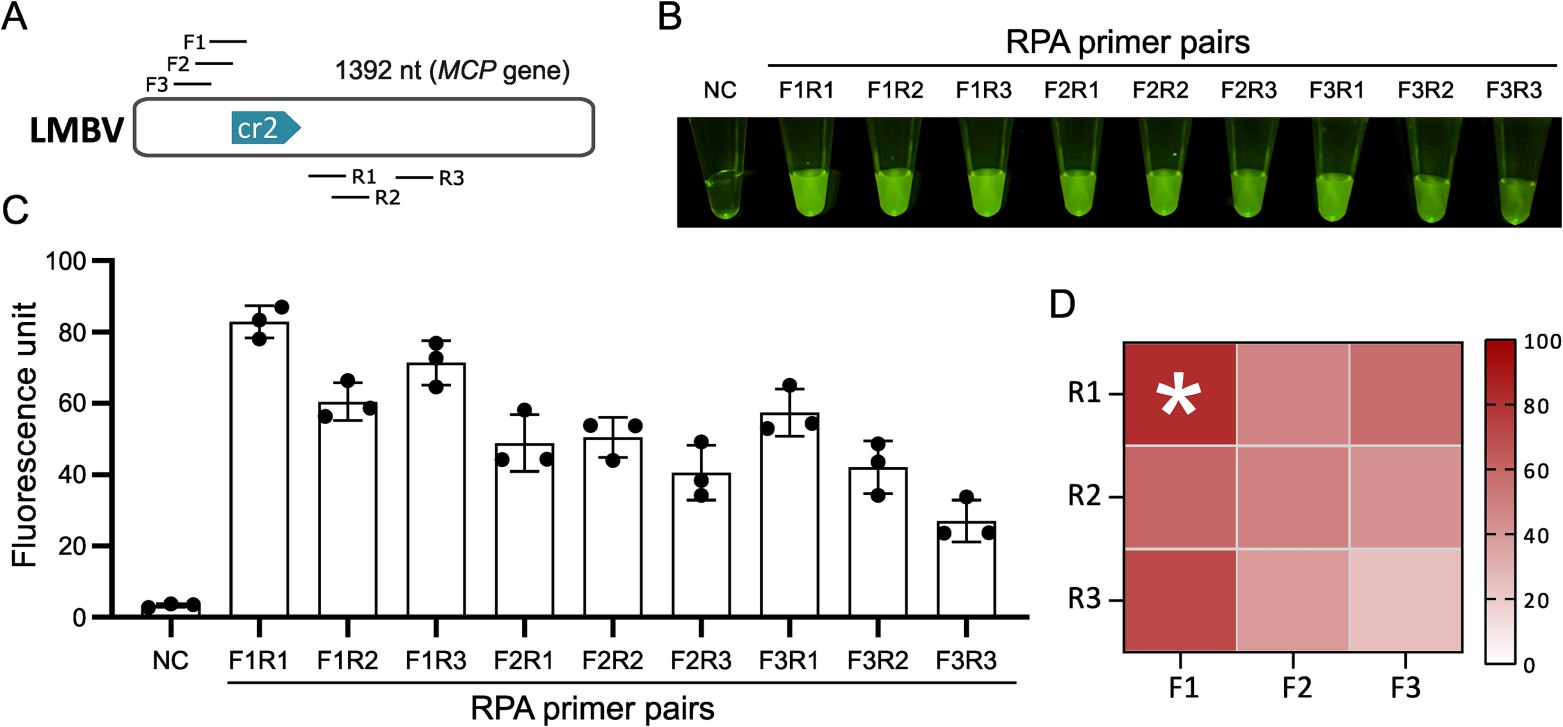
Screening of RPA primers. **(A)** Locations of candidate RPA primers targeting the *MCP* gene for the optimal crRNA. **(B)** CRISPR reaction results for different RPA primer combinations. **(C)** Fluorescence intensity statistics for CRISPR reactions corresponding to various RPA primer combinations. **(D)** Optimal primer screening data presented using a heat map assay. NC: negative control. The * denotes the group with the highest fluorescence intensity and does not represent statistical significance.

Using the optimal crRNA *MCP*-cr2 (Table 1), three pairs of RPA primers (Table 2) were designed and screened to optimize the pre-amplification efficiency of the *MCP* gene (Figure 3A). Each forward and reverse primer combination was evaluated for amplification performance. The results indicated that the primer pair F1-R1 exhibited the highest fluorescence intensity in all the primer pairs, making it the most efficient RPA primer pair for LMBV detection (Figures 3B–D). Thus, the primer pair F1-R1 was selected for the subsequent experiments.

### 3.3 Sensitivity and specificity of RPA-CRISPR/Cas12a for LMBV

**Figure 4:**
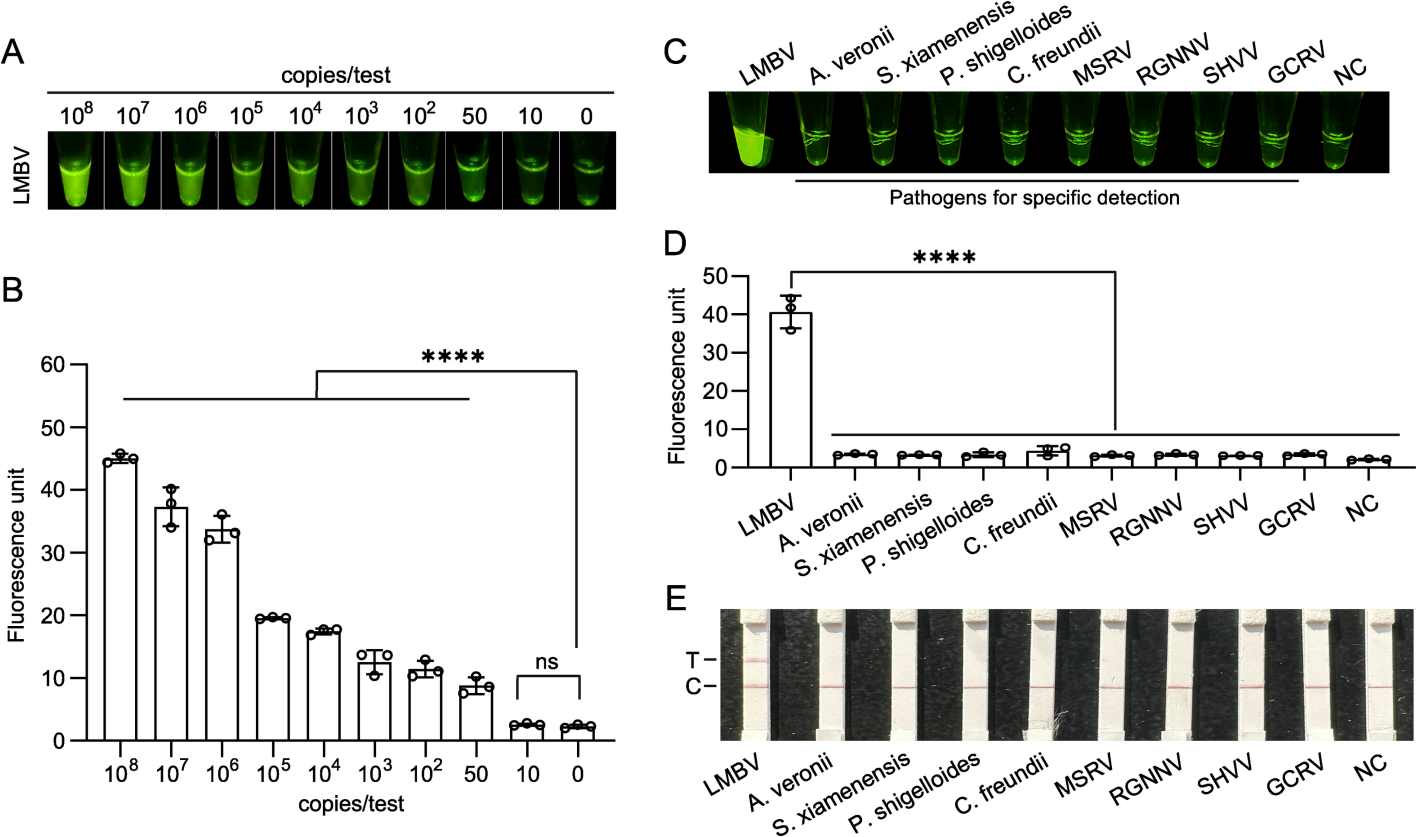
Sensitivity and specificity analysis of the Cas12a-based fluorescent method for LMBV detection. **(A,B)** Fluorescent intensity of the LMBV *MCP* gene detected via the CRISPR method at different plasmid concentrations, with results presented as naked-eye observations and microplate reader data. At least three technical replicates were performed for each biological sample. Error bars represent the standard error of three replicates. ****p < 0.0001, and ns, not signiticant. **(C,D)** Specificity analysis of the CRISPR method for LMBV detection against pathogens, including *A. veronii*, *S. xiamenensis*, *P. shigelloides*, *C. freundii*, MSRV, RGNNV, SHVV, and GCRV, with results shown as naked-eye observations and microplate reader data. NC represents the no-template controls. At least three technical replicates were performed for each biological sample. Error bars represent the standard error of three replicates. ****p < 0.0001. **(E)** Specificity assay results for templates with pathogens, including *A. veronii*, *S. xiamenensis*, *P. shigelloides*, *C. freundii*, MSRV, RGNNV, SHVV, and GCRV, using Lateral flow strips. T, test line; C, control line.

To assess the sensitivity of the RPA-CRISPR/Cas12a fluorescence assay, *MCP* gene templates with concentrations of 10^8^, 10^7^, 10^6^, 10^5^, 10^4^, 10^3^, 10^2^, 50, 10, and 0 copies per reaction were tested. The results of naked-eye observations and the microplate reader demonstrated that the limit of detection (LOD) for the *MCP* gene was 50 copies per reaction (Figures 4A,B).

For the specificity assay, fluorescence activity was observed exclusively with the LMBV DNA template. In contrast, DNA templates from other organisms, including *A. veronii, S. xiamenensis, P. shigelloides, C. freundii*, MSRV, RGNNV, SHVV, and GCRV, exhibited fluorescence levels comparable to the negative control, indicating no significant fluorescence in these reactions (Figures 4C,D). Furthermore, a distinct test band was observed on the Lateral flow strips, confirming that the visual detection system possesses high specificity (Figure 4E).

### 3.4 Clinical sample detection using the RPA-CRISPR/Cas12a based fluorescent method

**Figure 5:**
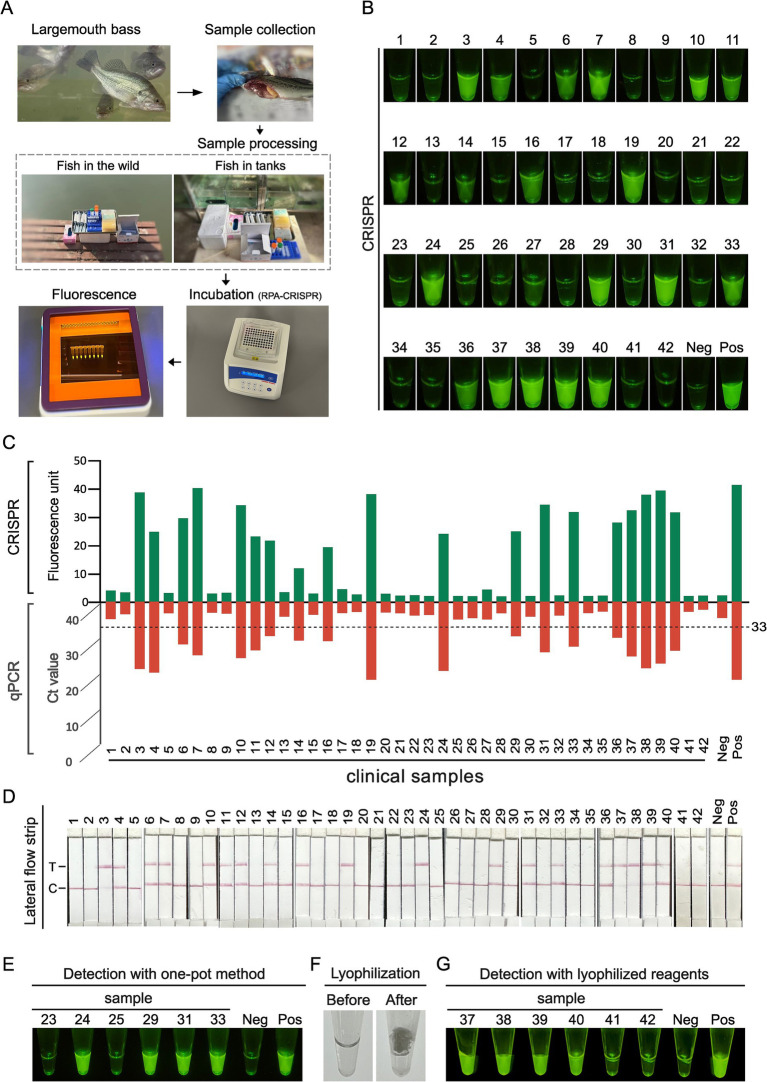
Clinical fish sample validation of RPA-CRISPR/Cas12a method. **(A)** Schematic diagram of the clinical nucleic acid detection process for LMBV. Sample collection for clinical testing can be conducted on-site, either in the field or near breeding tanks. The process begins with nucleic acid extraction, followed by amplification and enrichment using RPA methods. Finally, CRISPR-based fluorescence detection is used to determine the presence or absence of the target nucleic acids. **(B)** Results of RPA-CRISPR detection in 42 clinical fish samples indicated by green fluorescence. **(C)** The comparison of RPA-CRISPR detection and qPCR results in 42 clinical fish samples. **(D)** Detection of the LMBV in 42 clinical fish samples. T, test line; C, control line; Neg, negative control; Pos, positive control. **(E)** Detection with one-pot method using clinical samples. **(F)** Morphology of RPA-CRISPR reaction reagents before and after lyophilization. **(G)** Results of clinical samples were assayed using lyophilized RPA-CRISPR reaction reagents.

For clinical nucleic acid detection of LMBV, the process outlined in Figure 5A was implemented. Sample collection is carried out on-site, either in the field or near breeding tanks. Following nucleic acid extraction, the presence or absence of the target nucleic acids is determined using the RPA-CRISPR/Cas12a fluorescence detection method. A total of 42 fish liver samples were tested. To comprehensively assess the feasibility of the established assay for detecting LMBV samples, RPA-CRISPR/Cas12a and the gold standard qPCR were used for comparative validation. Both methods were repeated 3 times, and the consistency of the 2 methods was mutually analyzed. Among these, qPCR analysis identified 19 positive samples and 23 negative samples (Supplementary Table S1). The results of RPA-CRISPR/Cas12a fluorescence (Figure 5B) assay demonstrated a 100% concordance rate with the results of qPCR (Figure 5C) and LFA (Figure 5D). Notably, samples with low Ct values in the qPCR assay exhibited high fluorescence signals in the RPA-CRISPR/Cas12a method, indicating strong agreement and consistent performance between the different detection methods.

### 3.5 System optimization via integrated one-pot and lyophilization approaches

To facilitate the rapid application of the RPA-CRISPR method for on-site detection, we attempted a one-pot approach for sample detection. The results from six samples (Figure 5E) demonstrated that the one-pot method effectively detected target nucleic acids, yielding results consistent with the conventional two-step method. To improve detection system stability for field applications, we explored lyophilization strategy. Figure 5F shows the reaction mixture’s transition from liquid state before lyophilization to a near-microsphere state after freeze-drying, which enables more stable storage of the detection system. Testing six samples using this lyophilized reagent confirmed that the method effectively detected the target nucleic acids, with results consistent with the two-step method (Figure 5G). These findings demonstrate that both the one-pot approach and lyophilization strategy facilitate rapid and convenient detection of LMBV.

## 4 Discussion

Largemouth bass is a highly successful alien aquaculture species in China, but increasing stocking density and reduces genetic diversity have caused frequent disease outbreaks. Environmental fluctuations and intensive feeding further elevate mortality rates (Grant et al., 2003). Effective control of LMBV outbreaks requires early detection, facilitating timely prevention and treatment. While traditional PCR and qPCR methods relies on laboratory conditions and the inability to directly visualize PCR products limit their application for rapid, field-based detection (Arif et al., 2021). Thus, developing portable detection methods for LMBV is essential to enable early intervention and prevent viral spread.

The CRISPR-Cas system, uses crRNA and enzymes like Cas12 and Cas13 to target and cleave specific DNA or RNA sequences, is a powerful tool for pathogen detection with high specificity and sensitivity (Weng et al., 2023). For instance, a CRISPR/Cas13 method has been reported for LMBV detection (Guang et al., 2024). Within the CRISPR system, Cas12a and Cas13a perform trans-cleavage following *cis*-cleavage, but they differ in substrate specificity: Cas12a targets single-stranded DNA (ssDNA), while Cas13a targets single-stranded RNA (ssRNA) (Phuphisut et al., 2024). Cas13 can detect DNA, but requires an additional transcription step to convert the target into RNA. Furthermore, the ssRNA reporter used in Cas13-based assays may increase susceptibility to false-positive signals and presents stability challenges during storage (Li et al., 2022b). Cas12a is particularly valuable for its DNA-targeting nuclease activity, enabling direct DNA detection without requiring *in vitro* transcription (IVT) to RNA (Yang J. et al., 2022). Upon forming a Cas12a-crRNA complex with target DNA, Cas12a exhibits collateral cleavage activity on nonspecific ssDNA (Li et al., 2018; Nguyen et al., 2020). Thus, we chose Cas12a for development the LMBV detection method (Figure 1).

The development of precise CRISPR-based assays for viral detection requires systematic optimization. First, appropriate target genes must be selected to ensure assay specificity (Ahamed et al., 2024). Most PCR assays for LMBV detection target the conserved ranaviral *MCP* gene, which encodes a major capsid protein critical for the viral structure (Grizzle et al., 2003; Iwanowicz et al., 2013). As a highly conserved protein, MCP is essential for classifying iridoviruses and studying the evolutionary dynamics of key target genes in this viral family (Nakajima et al., 1998; Ohlemeyer et al., 2011).

Based on 19 available LMBV *MCP* gene sequences (Figure 2A), 5 specific crRNAs and 3 pairs of RPA primers were designed (Tables 1, 2). The Cas12a protein requires crRNAs to recognize sequences adjacent to the protospacer adjacent motif (PAM) within the target DNA template, initiating trans-cleavage of nonspecific ssDNA (Karvelis et al., 2020). Validation of the selected crRNAs and RPA primers confirmed the efficiency of the established method (Figures 2, 3). Another reporter, labeled with a FAM molecule at the 5′ end and biotin at the 3′ end, was also employed. In the specific assay, its degradation becomes visible to the naked eye when paired with a lateral flow strip, demonstrating potential for development as an alternative clinical detection method (Figure 3E).

Isothermal amplification technology (ITA) eliminates the reliance of traditional PCR on thermal cycling equipment, reducing testing costs and laboratory requirements, making it suitable for developing detection platforms (Wang et al., 2023). Among various isothermal amplification methods, RPA stands out for its simplicity, as it does not require temperature control or complex primer design. Integrating CRISPR/Cas12a with RPA enhancing the detection compatibility (Li et al., 2022b). These features make RPA-CRISPR/Cas12a especially suitable for field-based applications. We designed multiple pairs of forward and reverse primers on *MCP* gene and obtained optimal primer pair by RPA amplification (Figure 3).

The accuracy of the RPA-CRISPR/Cas12a assay was evaluated using 42 fish samples (Figures 5A–D), which highlights the reliability of this method for the clinical detection of LMBV. The conventional two-step detection method is somewhat cumbersome and carries potential contamination risks. Therefore, we further optimized our approach by developing a one-step assay to simplify the workflow and reduce detection time. The results demonstrate the efficiency of this one-pot RPA-CRISPR/Cas12a method for target detection (Figure 5E). Furthermore, lyophilization significantly reduces the reliance on cold-chain transport and extends the shelf life of the diagnostics, enhancing viability for use in low-resource and remote settings (Ahamed et al., 2024). The use of lyophilized reagents makes RPA particularly suitable for rapid nucleic acid testing (Shi et al., 2022; Shi et al., 2024). In our study, we confirmed the effectiveness of lyophilized one-pot RPA-CRISPR/Cas12a reagents for sample detection (Figures 5F,G).

Recent advances in pathogen detection, including Mpox virus diagnostics (Asif et al., 2024) and crRNA design optimization through tools like EasyDesign (Huang et al., 2024), incorporate artificial intelligence (AI) approaches spanning machine learning and deep learning techniques. AI-driven strategies accelerate diagnostic development for infectious diseases by efficiently identifying optimal nucleic acid targets, thereby reducing experimental validation requirements. The integration of AI with CRISPR-based diagnostics represents a paradigm shift in pathogen detection.

This innovative technique, based on the *MCP* gene, represents the first RPA-CRISPR/Cas12 method developed for LMBV detection. It showed no cross-amplification with other common aquaculture pathogens, ensuring specificity, while its high efficiency and sensitivity make it a reliable tool for fish farmers and government supervision departments to monitor LMBV in aquaculture, thereby helping to prevent the significant economic losses caused by disease outbreaks.

## 5 Conclusion

An RPA-CRISPR/Cas12a-based method for LMBV detection was developed, targeting the *MCP* gene with optimal crRNA and RPA primers. The highly specific method detects 50 copies of target nucleic acid within 40 min with no requirement of specialized equipment. Validation with clinical samples showed 100% concordance with qPCR and lateral flow strip assay. The convenient and stable application characteristics in clinical sample testing were enhanced by one-pot and lyophilization strategy. Our study offers a rapid, reliable tool for viral diagnosis and monitoring of LMBV infection.

## Data Availability

The datasets presented in this study can be found in online repositories. The names of the repository/repositories and accession number(s) can be found in the article/Supplementary material.
