# Supplementary material for: Development of a rapid on-site detection method for largemouth bass virus based on RPA-CRISPR/Cas12a system
Source: Front Microbiol. 2025 May 21;16:1599006. doi: 10.3389/fmicb.2025.1599006 (PMC12133836; doi:10.3389/fmicb.2025.1599006)
Supplement: Supplementary file 1 [file Data_Sheet_1.pdf]

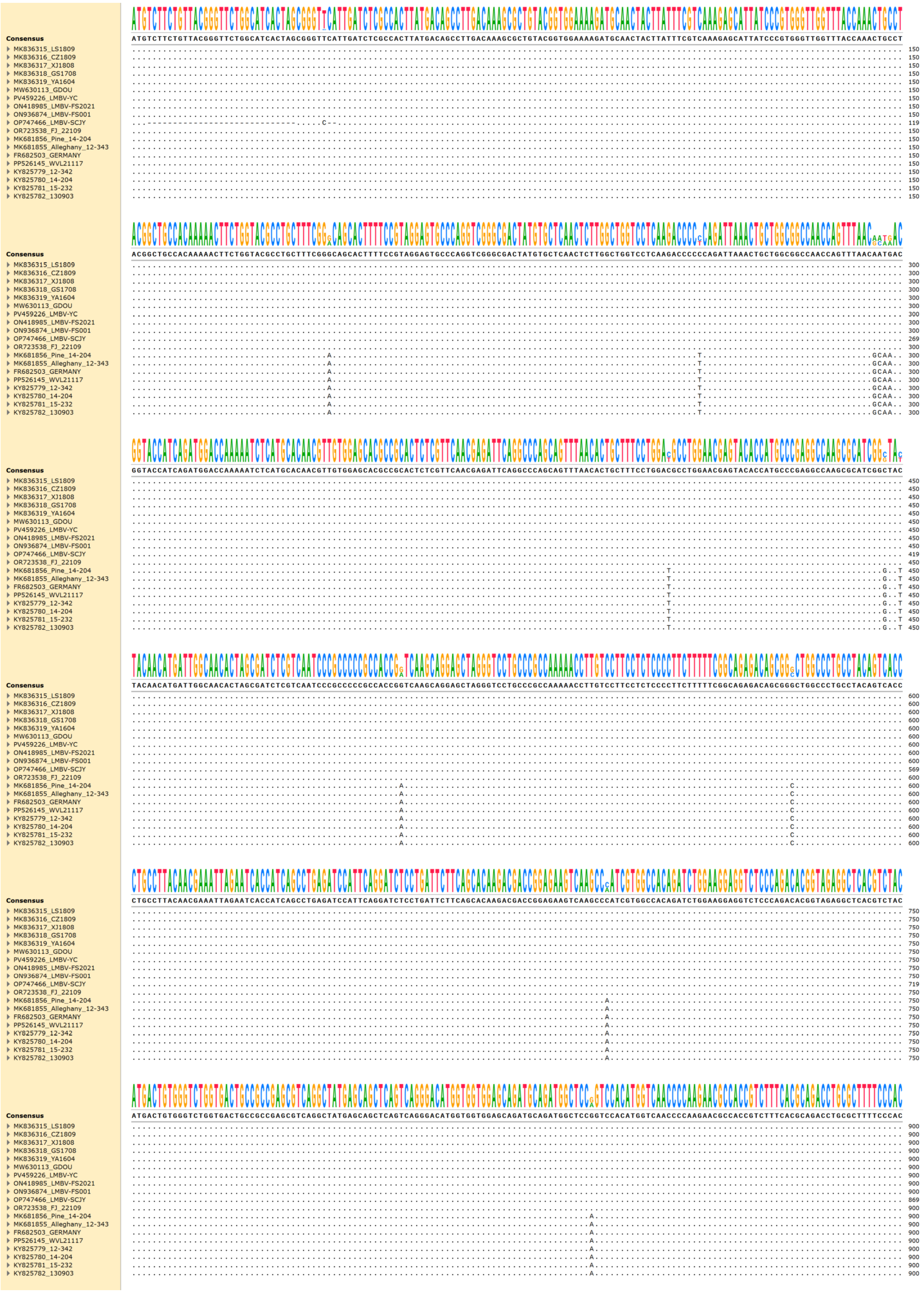
Consensus
MK836315_LS1809
MK836316_CZ1809
MK836317_XJ1808
MK836318_GS1708
MK836319_YA1604
MW630113_GDOU
PV459226_LMBV-YC
ON418985_LMBV-FS2021
ON936874_LMBV-FS001
OP747466_LMBV-SCJY
OR723538_FJ_22109
MK681856_Pine_14-204
MK681855_Alleghany_12-343
FR682503_GERMANY
PP526145_WVL21117
KY825779_12-342
KY825780_14-204
KY825781_15-232
KY825782_130903

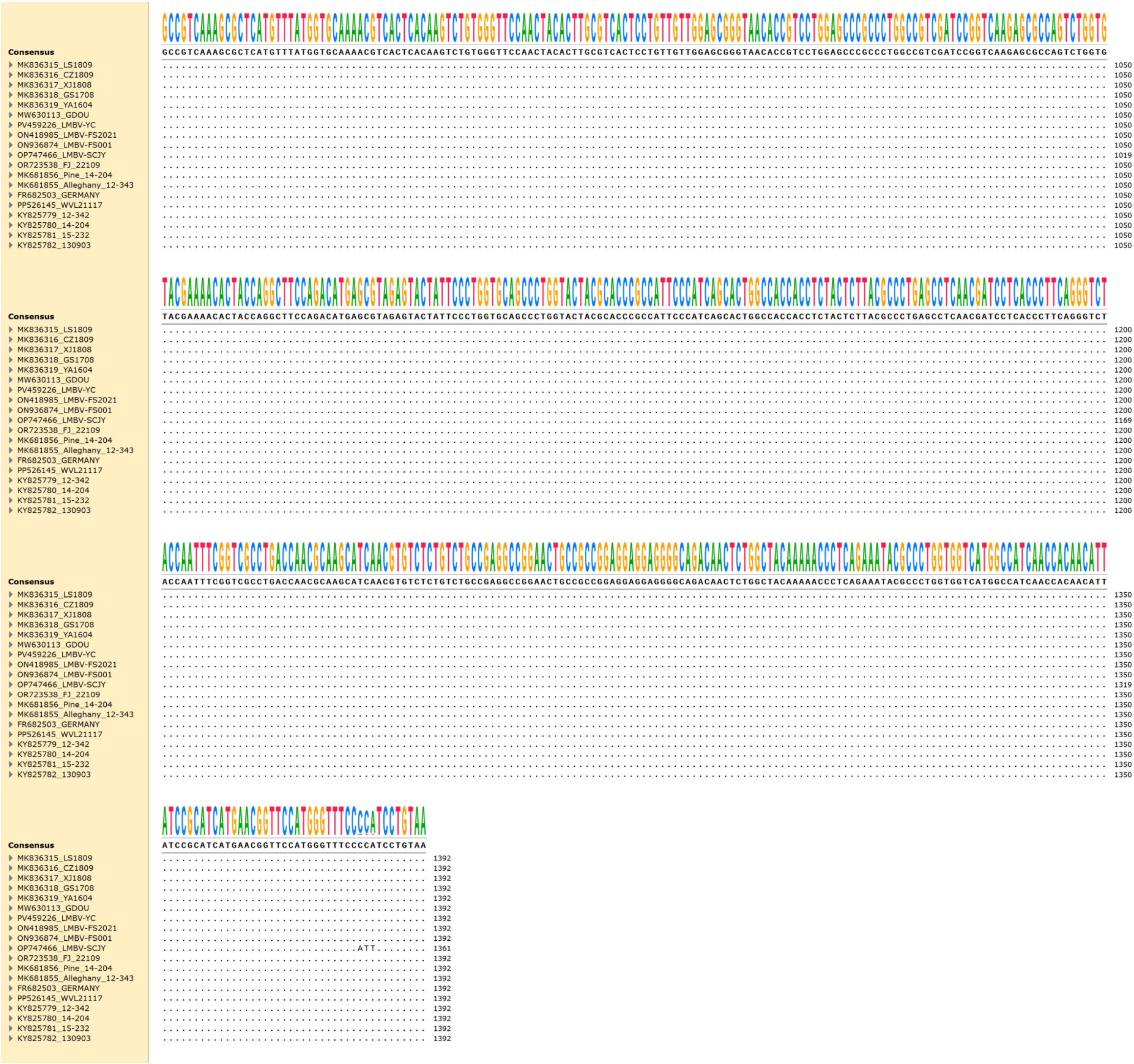
Consensus
GCCGTCAAAGCGCTCATGTTTATGGTGCAAAACGTCACTCACAAGTCTGTGGGTTCCAACTACACTTGCGTCACTCCTGTTGTTGGAGCGGGTAACACCGTCCTGGAGCCCGCCCTGGCCGTCGATCCGGTCAAGAGCGCCAGTCTGGTG
MK836315_LS1809 1050
MK836316_CZ1809 1050
MK836317_XJ1808 1050
MK836318_GS1708 1050
MK836319_YA1604 1050
MW630113_GDOU 1050
PV459226_LMBV-YC 1050
ON418985_LMBV-FS2021 1050
ON936874_LMBV-FS001 1050
OP747466_LMBV-SCJY 1019
OR723538_FJ_22109 1050
MK681856_Pine_14-204 1050
MK681855_Alleghany_12-343 1050
FR682503_GERMANY 1050
PP526145_WVL21117 1050
KY825779_12-342 1050
KY825780_14-204 1050
KY825781_15-232 1050
KY825782_130903 1050
Consensus
TACGAAAACACTACCAGGCTTCCAGACATGAGCGTAGAGTACTATTCCCTGGTGCAGCCCTGGTACTACGCACCCGCCATTCCCATCAGCACTGGCCACCACCTCTACTCTTACGCCCTGAGCCTCAACGATCCTCACCCTTCAGGGTCT
MK836315_LS1809 1200
MK836316_CZ1809 1200
MK836317_XJ1808 1200
MK836318_GS1708 1200
MK836319_YA1604 1200
MW630113_GDOU 1200
PV459226_LMBV-YC 1200
ON418985_LMBV-FS2021 1200
ON936874_LMBV-FS001 1200
OP747466_LMBV-SCJY 1169
OR723538_FJ_22109 1200
MK681856_Pine_14-204 1200
MK681855_Alleghany_12-343 1200
FR682503_GERMANY 1200
PP526145_WVL21117 1200
KY825779_12-342 1200
KY825780_14-204 1200
KY825781_15-232 1200
KY825782_130903 1200
Consensus
ACCAATTTCGGTCGCCTGACCAACGCAAGCATCAACGTGTCTCTGTCTGCCGAGGCCGGAACTGCCGCCGGAGGAGGAGGGGCAGACAACTCTGGCTACAAAAACCCTCAGAAATACGCCCTGGTGGTCATGGCCATCAACCACAACATT
MK836315_LS1809 1350
MK836316_CZ1809 1350
MK836317_XJ1808 1350
MK836318_GS1708 1350
MK836319_YA1604 1350
MW630113_GDOU 1350
PV459226_LMBV-YC 1350
ON418985_LMBV-FS2021 1350
ON936874_LMBV-FS001 1350
OP747466_LMBV-SCJY 1319
OR723538_FJ_22109 1350
MK681856_Pine_14-204 1350
MK681855_Alleghany_12-343 1350
FR682503_GERMANY 1350
PP526145_WVL21117 1350
KY825779_12-342 1350
KY825780_14-204 1350
KY825781_15-232 1350
KY825782_130903 1350
Consensus
ATCCGCATCATGAACGGTTCCATGGGTTTCCCCATCCTGTAA
MK836315_LS1809 1392
MK836316_CZ1809 1392
MK836317_XJ1808 1392
MK836318_GS1708 1392
MK836319_YA1604 1392
MW630113_GDOU 1392
PV459226_LMBV-YC 1392
ON418985_LMBV-FS2021 1392
ON936874_LMBV-FS001 1392
OP747466_LMBV-SCJY ATT 1361
OR723538_FJ_22109 1392
MK681856_Pine_14-204 1392
MK681855_Alleghany_12-343 1392
FR682503_GERMANY 1392
PP526145_WVL21117 1392
KY825779_12-342 1392
KY825780_14-204 1392
KY825781_15-232 1392
KY825782_130903 1392
