# Supplementary material for: Development of a rapid on-site detection method for largemouth bass virus based on RPA-CRISPR/Cas12a system
Source: Front Microbiol. 2025 May 21;16:1599006. doi: 10.3389/fmicb.2025.1599006 (PMC12133836; doi:10.3389/fmicb.2025.1599006)
Supplement: Supplementary file 2 [file Data_Sheet_2.docx]

**Table S1. qPCR results of clinical LMBV samples.**

| Sample ID | Ct value in qPCR | | | P/N | Mean | SD | Sample ID | Ct value in qPCR | | | P/N | Mean | SD |
| --- | --- | --- | --- | --- | --- | --- | --- | --- | --- | --- | --- | --- | --- |
| 1 | 34.74 | 34.11 | 33.71 | N | 34.19 | 0.52 | 23 | 35.42 | 34.70 | 35.86 | N | 35.33 | 0.59 |
| 2 | 33.23 | 33.23 | 33.36 | N | 33.27 | 0.08 | 24 | 19.49 | 19.60 | 19.64 | P | 19.58 | 0.08 |
| 3 | 19.03 | 20.63 | 20.72 | P | 20.13 | 0.95 | 25 | 34.13 | 33.23 | 33.56 | N | 33.64 | 0.46 |
| 4 | 19.10 | 19.36 | 18.89 | P | 19.12 | 0.24 | 26 | 34.33 | 34.50 | 34.57 | N | 34.46 | 0.12 |
| 5 | 34.07 | 33.21 | 32.70 | N | 33.33 | 0.69 | 27 | 35.49 | 35.67 | 35.80 | N | 35.65 | 0.15 |
| 6 | 27.15 | 27.73 | 26.24 | P | 27.04 | 0.75 | 28 | 35.71 | 35.99 | 35.77 | N | 35.83 | 0.15 |
| 7 | 23.98 | 24.11 | 24.02 | P | 24.04 | 0.06 | 29 | 29.44 | 29.34 | 29.22 | P | 29.33 | 0.11 |
| 8 | 35.97 | 35.99 | 35.91 | N | 35.96 | 0.04 | 30 | 35.07 | 34.60 | 34.93 | N | 34.87 | 0.24 |
| 9 | 35.48 | 35.75 | 35.85 | N | 35.70 | 0.19 | 31 | 24.94 | 24.53 | 25.05 | P | 24.84 | 0.27 |
| 10 | 23.39 | 22.94 | 23.20 | P | 23.18 | 0.23 | 32 | 34.74 | 34.81 | 35.93 | N | 35.16 | 0.67 |
| 11 | 25.27 | 25.87 | 25.05 | P | 25.40 | 0.43 | 33 | 26.48 | 26.58 | 26.31 | P | 26.45 | 0.14 |
| 12 | 29.23 | 29.66 | 29.41 | P | 29.43 | 0.22 | 34 | 35.35 | 36.18 | 36.06 | N | 35.86 | 0.45 |
| 13 | 35.74 | 34.68 | 34.12 | N | 34.84 | 0.83 | 35 | 36.13 | 36.33 | 36.51 | N | 36.33 | 0.19 |
| 14 | 28.27 | 28.17 | 28.04 | P | 28.16 | 0.11 | 36 | 29.20 | 28.93 | 28.68 | P | 28.93 | 0.26 |
| 15 | 35.15 | 35.94 | 35.12 | N | 35.40 | 0.47 | 37 | 23.53 | 23.81 | 23.73 | P | 23.69 | 0.15 |
| 16 | 27.87 | 28.00 | 28.02 | P | 27.96 | 0.08 | 38 | 20.06 | 20.54 | 20.54 | P | 20.38 | 0.28 |
| 17 | 35.89 | 36.02 | 35.77 | N | 35.89 | 0.13 | 39 | 22.26 | 22.25 | 22.32 | P | 22.28 | 0.04 |
| 18 | 36.23 | 36.54 | 35.73 | N | 36.17 | 0.41 | 40 | 25.26 | 25.49 | 25.07 | P | 25.27 | 0.21 |
| 19 | 17.14 | 16.83 | 17.27 | P | 17.08 | 0.22 | 41 | 35.54 | 35.53 | 35.59 | N | 35.55 | 0.03 |
| 20 | 35.87 | 36.25 | 35.98 | N | 36.03 | 0.20 | 42 | 36.07 | 35.19 | 35.94 | N | 35.73 | 0.47 |
| 21 | 35.53 | 36.07 | 35.78 | N | 35.79 | 0.27 | N control | 34.75 | 34.29 | 34.42 |  | 34.48 | 0.24 |
| 22 | 34.55 | 35.66 | 35.37 | N | 35.19 | 0.58 | P control | 16.90 | 16.94 | 17.45 |  | 17.10 | 0.31 |

Note: “P”, positive; “N”, negative.

**Table S2.** **The nucleotide sequence of LMBV *MCP* gene.**

| **Target gene** | **Sequence** |
| --- | --- |
| *Micropterus salmoides*  LMBV-*MCP*  ON418985  1524 bp | AATAAAAGGAATGTCTTCTGTTACGGGTTCTGGCATCACTAGCGGGTTCATTGATCTCGCCACTTATGACAGCCTTGACAAAGCGCTGTACGGTGGAAAAGATGCAACTACTTATTTCGTCAAAGAGCATTATCCCGTGGGTTGGTTTACCAAACTGCCTACGGCTGCCACAAAAACTTCTGGTACGCCTGCTTTCGGGCAGCACTTTTCCGTAGGAGTGCCCAGGTCGGGCGACTATGTGCTCAACTCTTGGCTGGTCCTCAAGACCCCCCAGATTAAACTGCTGGCGGCCAACCAGTTTAACAATGACGGTACCATCAGATGGACCAAAAATCTCATGCACAACGTTGTGGAGCACGCCGCACTCTCGTTCAACGAGATTCAGGCCCAGCAGTTTAACACTGCTTTCCTGGACGCCTGGAACGAGTACACCATGCCCGAGGCCAAGCGCATCGGCTACTACAACATGATTGGCAACACTAGCGATCTCGTCAATCCCGCCCCCGCCACCGGTCAAGCAGGAGCTAGGGTCCTGCCCGCCAAAAACCTTGTCCTTCCTCTCCCCTTCTTTTTCGGCAGAGACAGCGGGCTGGCCCTGCCTACAGTCACCCTGCCTTACAACGAAATTAGAATCACCATCAGCCTGAGATCCATTCAGGATCTCCTGATTCTTCAGCACAAGACGACCGGAGAAGTCAAGCCCATCGTGGCCACAGATCTGGAAGGAGGTCTCCCAGACACGGTAGAGGCTCACGTCTACATGACTGTGGGTCTGGTGACTGCCGCCGAGCGTCAGGCTATGAGCAGCTCAGTCAGGGACATGGTGGTGGAGCAGATGCAGATGGCTCCGGTCCACATGGTCAACCCCAAGAACGCCACCGTCTTTCACGCAGACCTGCGCTTTTCCCACGCCGTCAAAGCGCTCATGTTTATGGTGCAAAACGTCACTCACAAGTCTGTGGGTTCCAACTACACTTGCGTCACTCCTGTTGTTGGAGCGGGTAACACCGTCCTGGAGCCCGCCCTGGCCGTCGATCCGGTCAAGAGCGCCAGTCTGGTGTACGAAAACACTACCAGGCTTCCAGACATGAGCGTAGAGTACTATTCCCTGGTGCAGCCCTGGTACTACGCACCCGCCATTCCCATCAGCACTGGCCACCACCTCTACTCTTACGCCCTGAGCCTCAACGATCCTCACCCTTCAGGGTCTACCAATTTCGGTCGCCTGACCAACGCAAGCATCAACGTGTCTCTGTCTGCCGAGGCCGGAACTGCCGCCGGAGGAGGAGGGGCAGACAACTCTGGCTACAAAAACCCTCAGAAATACGCCCTGGTGGTCATGGCCATCAACCACAACATTATCCGCATCATGAACGGTTCCATGGGTTTCCCCATCCTGTAAATTCAACACATATTACTACGTTTCCATATCGCAAACTGCGATATGGAAAAACTTGAACGCCCCTCTTGTTAGTTTCTGTCACCTAAATTCGTTTTTACACAAAACAACGCAAATGGCAAGGC |

**Table S3. LMBV isolates used in phylogenetic and genetic analyses: strain names, abbreviations, and GenBank accession numbers.**

| **Isolate name** | **Abbreviation** | **GenBank accession number** |
| --- | --- | --- |
| Largemouth bass virus isolate Santee-Cooper Reservoir | LMBV- SC95 | FR682503.1 |
| Largemouth bass virus isolate LMBIV_Kn_460_03 | LMBIV-Kn-460-03 | JF264364.1 |
| Largemouth bass virus isolate Alleghany 12-343 | LMBV Alleghany 12-343 | MK681855.1 |
| Largemouth bass virus isolate Pine 14-204 | LMBV-Pine 14-204 | MK681856.1 |
| Largemouth bass virus isolate LS1809 | LMBV-LS1809 | MK836315.1 |
| Largemouth bass virus isolate CZ1809 | LMBV-CZ1809 | MK836316.1 |
| Largemouth bass virus isolate XJ1808 | LMBV-XJ1808 | MK836317.1 |
| Largemouth bass virus isolate GS1708 | LMBV-GS1708 | MK836318.1 |
| Largemouth bass virus isolate YA1604 | LMBV-YA1604 | MK836319.1 |
| Largemouth bass virus isolate Ningbo | LMBV-Ningbo | MN176304.1 |
| Largemouth bass virus isolate LMBV-FS001 | LMBV-FS001 | OM319463.1 |
| Largemouth bass virus isolate LMBV-FS2021 | LMBV-FS2021 | ON418985.1 |
| Largemouth bass virus isolate LMBV-SCJY | LMBV-SCJY | OP747466.1 |
| Largemouth bass virus strain FJ_22109 | LMBV-FJ22109 | OR723538.1 |
| Largemouth bass virus isolate LMBV-YC | LMBV-YC | PV459226.1 |
| Smallmouth bass virus isolate 12-342 | SMBV-12-324 | KY825779.1 |
| Smallmouth bass virus isolate 14-204 | SMBV-14-204 | KY825780.1 |
| Smallmouth bass virus isolate 15-232 | SMBV-15-232 | KY825781.1 |
| Smallmouth bass virus isolate 130903 | SMBV-130903 | KY825782.1 |

**Table S4.** **Target region and primer sequences for qPCR detection of LMBV *MCP* gene**

| **Target gene** | **Sequence** |
| --- | --- |
| q*MCP*-F | 5′-TCTCGCCACTTATGACAGCC-3′ |
| q*MCP*-R | 5′-AGTTGAGCACATAGTCGCCC-3′ |
| *MCP* gene target region | TCTCGCCACTTATGACAGCCTTGACAAAGCGCTGTACGGTGGAAAAGATGCAACTACTTATTTCGTCAAAGAGCATTATCCCGTGGGTTGGTTTACCAAACTGCCTACGGCTGCCACAAAAACTTCTGGTACGCCTGCTTTCGGGCAGCACTTTTCCGTAGGAGTGCCCAGGTCGGGCGACTATGTGCTCAACT |
